# Interventions regarding physicians’ sickness certification practice – a systematic literature review with meta-analyses

**DOI:** 10.1080/02813432.2022.2036420

**Published:** 2022-03-07

**Authors:** Söderman M., Wennman-Larsen A., Hoving J. L., Alexanderson K., Friberg E.

**Affiliations:** a Mälardalen University, Eskilstuna, Sweden; b Division of Insurance Medicine, Department of Clinical Neuroscience, Karolinska Institutet, Stockholm, Sweden; c Sophiahemmet University, Stockholm, Sweden; d Amsterdam UMC, location Academic Medical Center, University of Amsterdam, Department of Public and Occupational Health, Amsterdam Public Health research institute, and Research center for Insurance Medicine, Amsterdam, The Netherlands

**Keywords:** Sick leave, return to work, physicians’ practice patterns, sickness certification, intervention, insurance medicine, systematic review, meta-analysis, CRD42019119697 (a revised protocol is under assessment after submission 4 September 2020, delay due to the covid-19 situation)

## Abstract

**Objective:**

A variety of interventions aiming to influence physicians’ sickness certification practice have been conducted, most are, however, not evaluated scientifically. The aim of this systematic literature review was to obtain updated knowledge about interventions regarding physicians’ sickness certification practice and to summarize their possible effects, in terms of sickness absence (SA) or return to work (RTW) among patients.

**Methods:**

We searched PubMed and Web of Science up through 15 June 2020 and selected peer-reviewed studies that reported effects of controlled interventions that aimed to improve physicians’ sickness certification practice and used SA or RTW among patients as outcome measures. Meta-analyses were conducted using random-effect models.

**Results:**

Of the 1399 identified publications, 12 studies covering 9 interventions were assessed as relevant and included in the review. Most (70%) were from the Netherlands, two had a controlled, and seven a randomized controlled study design. All interventions included some type of training of physicians, and two interventions also included IT-support. Regarding the outcomes of SA/RTW, 30 different effect measures were used. In the meta-analyses, no statistically significant effect in favor of the interventions was observed for having any RTW (i.e. first, partial, or full) nor full RTW.

**Conclusions:**

The individual studies showed that physicians’ sickness certification practice might be influenced by interventions in both the intended and non-intended direction, however, no statistically significant effect was indicated by the meta-analysis. The included studies varied considerably concerning intervention content and effect measures.

**KEY POINTS**
- The knowledge is very limited regarding the content of interventions directed to physician’s sickness certification practice
- The identified interventions included some type of training of physicians, and some of them also included IT-support for physicians
- There was a great heterogeneity among the interventions concerning effect measures used regarding return to work among patients
- The individual studies showed that physicians’ sickness certification practice might be influenced by interventions in both intended and non-intended directions, however, the overall meta-analysis did not indicate an effect.

## Introduction

Physician’s sickness certification of patients in need of sickness absence (SA) is a healthcare task with impact both on patients, employers, healthcare, insurers, authorities, and the society as a whole. SA incurs large costs to society, including costs for productivity loss, healthcare, and efforts to get the sickness absentee back to work [1]. Possible side effects from being on SA, such as other types of morbidity, higher morbidity and mortality, or reduced wellbeing, have also been discussed [2–6]. Moreover, studies have shown the importance of having paid work for health in general, also specifically with regard to RTW after SA [7,8].

As physicians in most countries have a central role in the sickness certification process, a variety of interventions aiming to improve physician’s sickness certification practices have been implemented in many western countries. However, most of these interventions have not been evaluated scientifically [9,10]. Sickness certification for SA is a rather common task among physicians [11] and includes assessment of whether the disease or injury has resulted in functional limitations reducing the patient’s work capacity in relation to his or hers work demands [9,12–17]. Physicians are also to discuss the pros and cons of SA with the patient and, if agreed on SA, consider its duration, grade (full- or part-time SA), the need of referrals to and collaboration with others within and outside healthcare, make a plan for actions to take place during the SA, write a sickness certificate, and document the actions taken [9,15,18,19]. According to previous reviews, physicians find five areas of sickness certification problematic, namely: the assessment of patients’ work capacity, a lack of competence (i.e. knowledge, skills, and attitudes) concerning sickness certification/insurance medicine, problems in handling the two roles as the patient’s treating physician and as a medical expert writing certificates, managing disagreements with patients regarding the need for SA, and cooperating with other actors in SA cases [9–11]. In addition, political decisions on laws and regulations for medical SA can be aggravating aspects for physician’s sickness certification practice [20]. Physicians have also expressed the need for more competence and organisational requisites, including support regarding management of sickness certification tasks [16,21–24].

In a previous systematic review by The Swedish Agency on Health Technology Assessment [10] about physicians’ sickness certification practices including studies published until October 2002, only four intervention studies were assessed as having high enough quality to be included. That review was later followed-up by another review on the same topic, with studies published until August 2009 where three additional interventions of high enough quality were identified [9].

Although these studies have described some interventions that aim to improve how physicians handle sickness certification of patients, the knowledge is very limited regarding the content and effect of such interventions, e.g. regarding SA or RTW of patients. In this study we, therefore, focused on characteristics and possible effects of such interventions in terms of SA and RTW among patients.

## Objectives

The aim was to obtain updated knowledge about interventions regarding physicians’ sickness certification practice and summarize their possible effects, in terms of SA or RTW among their patients.

## Methods

A review protocol was added in the International prospective register of systematic reviews in health and social care (PROSPERO), registration number CRD42019119697 (update 4 September 2020), following the Preferred Reporting Items for Systematic review and Meta-Analysis Protocols (PRISMA-P) 2015s checklist [25].

### Inclusion criteria

In this review, interventions with effects measured as SA or RTW (or other comparable concepts) among patients were included. The other main group of outcome measures mentioned in the study protocol in PROSPERO, i.e. the physicians-related measures, will be reported elsewhere.

The following inclusion criteria were used in this review; controlled experimental studies including interventions regarding physicians’ sickness certification practice, presenting information about the physicians (e.g. number of physicians, physicians’ speciality), and intervention effects measured as SA or RTW among patients, and published in English in a scientific journal after peer review 1 January 2009 through 10 March 2018, and updates with added search terms 1 January 2009 through 15 June 2020. In addition to these searches, studies identified in two previous relevant literature reviews were included if they fulfilled our inclusion criteria [9,10].

### Information sources and search strategies

The search terms were formulated in accordance to Population, Intervention, Comparator, Outcome (PICO) framework (Supplementary Appendix, table 1) [26], and the complete search string for each database is presented in Supplementary Appendix, table 1. The searches followed well-established recommendations for systematic reviews and meta-analyses [27].

**Table 1.** The outcomes measures regarding sickness absence (SA) and return to work (RTW) used in the nine included intervention studies regarding physicians’ sickness certification.

Time to RTW
First RTW, from the first day of SA to the first day of RTW, measured as number of workers [36]^a^
Time to first RTW, in days from randomization to RTW [41]^a^
Days to partial RTW, in the same job as before the onset of the SA or a job with equal earnings [32,33]^a^
Number of days on SA between inclusion to the intervention and RTW to previous job without reduction of duties [37]^a^
Work resumption rate as dependent/non-dependent of SA benefits [39]^a^
Mean of total hours of SA before RTW [36]^a^
Mean days for patients’ SA episodes [35]
Median number of SA days from first day of SA until lasting (at least four weeks) full RTW [30]
Number of workers with full RTW, from the first day of SA to the first day of full RTW [36]^a^
Mean days to full RTW, from the first day of SA to the first day of full RTW [36]^a^
Median of days to full RTW, from the first day of SA to the first day of full RTW [36]^a^
Mean days to first RTW, from the first day of SA to the first day of full RTW [36]^a^
Median of days to first RTW, from the first day of SA to the first day of full RTW [36]^a^
Time in days to full lasting RTW from onset of SA to RTW at 3 months follow-up [38]
Time in days to full lasting RTW from onset of SA to RTW at 6 months follow-up [38]
Number of calendar days to full RTW, contracted working hours/week from the first day of SA to the first day of full RTW [40]^a^
Days to full RTW, in the same job as before the onset of the SA or a job with equal earnings [32,33]^a^
Time to full RTW, in days from randomization to RTW [41]^a^
**Estimates for probability of SA or RTW measures**
HR for first day of full RTW [36]^a^
HR for first day of RTW [36,41]^a^
HR for partial RTW [32,33,40]^a^
OR for part-time SA [35]^a^
HR for full RTW [32,33,40,41]^a^
HR for lasting full RTW [30]^a^
RR for lasting full RTW [38]
HR for RTW to previous job without reduction of duties [37]^a^
RR for dependent/non-dependent of SA benefits [39]^a^
HR for duration of patient SA episodes [35]^a^
HR for decreased prescription of active SA [35]^a^
RR for gradual dependent/non-dependent of SA benefits [39]^a^

SA: sickness absence; RTW: return to work; HR: hazard ratio; OR: odds ratio; RR: relative risk. ^a^Register data.

Publications were searched for in the following five ways:

PubMed and Web of Science through February 2019.

Communication with other researchers active within this research area about potentially relevant studies.
Reference tracking.
Citation tracking.
Studies identified in two previous relevant literature reviews [9,10].

### Assessment for relevance

**Figure 1. Flow chart showing selection of included studies.**
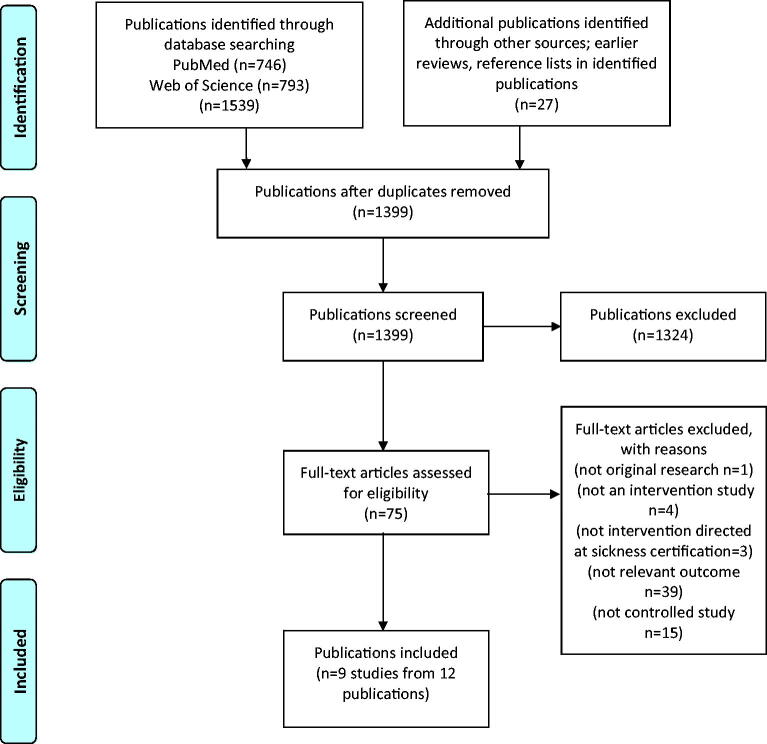


All identified publications were assessed for relevance according to the above-mentioned inclusion criteria. The studies were included regardless of degree of quality since a specific quality level was not an inclusion criterion in this review. Also, publications that were not included in the two previous reviews mentioned above, were assessed for relevance in the current review [9,10]. In Figure 1, the selection process is presented with a flow chart in accordance with PRISMA [28]. Using “Rayyan QCRI” software [29], screening was conducted independently and blinded by two of the authors (MS, EF) for search hits at a title and abstract level. Then, full-text screening of publications presenting relevant outcomes was conducted. Any discrepancies were resolved by consulting a third author (AWL) to reach an agreement. If a reviewer had co-authored any of the identified publications, someone else in the project group assessed that publication.

We checked if there was more than one publication from the same intervention study, among the publications deemed relevant for this review. This was the case for three of the interventions, two from the Netherlands [30–33] and one from Norway [34,35]. All these publications were included since they contributed with relevant information about the intervention that otherwise was missing.

### Data extraction

Data from the included interventions was extracted based on a template used in previous reviews of this type [9,10]. Aspects extracted are presented in the headings of Supplementary Appendix, table 2. Extraction of data was performed independently (by MS and EF or AWL), and disagreements were addressed *via* discussion among all authors until consensus was reached.

**Table 2.** Summarized relative risk estimates from meta-analyses using random effects model and 95% confidence intervals for interventions on physicians’ sickness certification practice measured as *any return to work (RTW) (first-, partial- or full RTW)* or as *full RTW*, stratified by type of intervention, type of physician, and geographical area.

	Studies (*n* = 9)	RR *any RTW* (95% CI)	*p* value for heterogeneity	*I^2^* statistic (%)	RR *full RTW* (95% CI)	*p* value for heterogeneity	*I^2^* statistic (%)
Type of intervention							
“Simple interventions”	7	1.03 (0.90–1.18)	0.019	60.4	0.96 (0.87–1.05)	0.075	47.6
“Complex interventions”	2	1.12 (0.73–1.74)	0.073	68.8	0.86 (0.37–1.98)	0.007	86.4
Design							
RCT	7	1.09 (0.99–1.19)	0.197	30.3	0.96 (0.86–1.07)	0.129	39.4
CT	2	1.74 (0.32–1.71)	0.083	66.7	0.76 (0.39–1.48)	0.004	87.9
Physicians							
OPs targeted (*n* = 297)	6	1.07 (0.98–1.16)	0.352	10.0	0.99 (0.88–1.11)	0.151	38.2
GPs targeted (*n* = 159)	3	1.95 (0.64–1.42)	0.001	84.7	0.86 (0.66–1.11)	0.016	75.7
Geographical area							
Netherlands	7	1.01 (0.88–1.15)	0.021	59.7	0.92 (0.78–1.10)	0.017	61.2
Rest of Europe	2	1.32 (1.06–1.65)	0.879	0.0	0.96 (0.83–1.11)	0.068	70.0

RR: relative risk; RCT: randomised controlled trial; CT: controlled trial; OP: occupational physician; GP: general practitioner.

### Data synthesis and statistical analyses

Characteristics of the included interventions, as well as the outcomes used within each intervention, were summarized in tables.

The meta-analyses were performed with data pooled using random-effects models. For the meta-analysis of SA or RTW, the outcomes in terms of relative risks (RR), odds ratios (OR), or hazard ratios (HR), and 95% confidence intervals (CI) for each intervention were used. For interventions not reporting such risk estimates, RRs were calculated from reported number of patients who no longer were on SA or who had returned to work, in both the intervention group (IG) and the control group (CG). Missing CIs were calculated using the presented p-values. In the meta-analyses, the effect measures were referred to as RTW, and these included both risk estimates calculated from presence of SA and time to RTW. Statistical heterogeneity between study-specific estimates was indicated with Cochran’s Q-test and the I^2^ statistics (a higher value indicated a greater degree of heterogeneity), and also by chi-squared tests (x^2^) presenting degrees of freedom (df) and probability with p-values (p). Stratified analyses were conducted for type of intervention (“simple” i.e. the intervention targeting only physicians, or “complex” interventions i.e. the intervention targeted both physicians and patients); design (randomized controlled trial (RCT) or controlled trial (CT)); type of physician targeted (occupational physician (OP), general practitioner (GP), or social insurance physician); and geographical area (Netherlands, rest of Europe (i.e. Belgium and Norway)). A sensitivity analysis was done by excluding one study at a time and then pooling the estimates for the rest of the studies. The meta-analyses were conducted using STATA 12, and the results were summarized in forest plots and in a table.

## Results

The searches resulted in 1399 unique publications. After screening of titles and abstracts, 1326 were excluded, resulting in 75 full-text publications assessed for relevance (Figure 1). Of these, 63 were excluded due to not being: original research, an intervention, an intervention targeting sickness certification practice, a controlled intervention, or not presenting relevant outcomes (that is, aspects of SA or RTW among the patients). The remaining 12 publications [30–41], from nine unique interventions, were included (Supplementary Appendix, table 1). A majority (seven out of nine interventions) of the included interventions were conducted in the Netherlands [30–33,36–38,40,41]. In total, 464 physicians were involved in the included interventions. In the Netherlands, most of the physicians were working as occupational physicians at occupational health services (*n* = 297) [32,33,36–41] and some were GPs (*n* = 46) [30,31] specifically handling sickness absentees (including screenings, referrals, and RTW efforts). In other countries, GPs in primary healthcare (*n* = 106) [34,35,39] or social insurance physicians (*n* = 15) [39], were targeted. For the outcomes SA or RTW, 6041 patients [30–33,36–41] and 2170 SA spells [34,35], respectively, were included.

### Description of interventions

Most of the interventions were directed towards physicians whose patients were on SA or were about to be sickness certified, with SA spells starting from the time of the intervention onset [40], or patients already on SA since up to three months [30,31,37]. In six interventions, the SA diagnosis targeted was mental disorders [30–33,36,38,40,41], in one, low-back pain [37], and in the other two the SA diagnosis were not specified [34,35,39]. Two of the nine intervention studies were designed as CTs [37,39] and seven as RCTs [30–36,38,40,41]. The control groups were either in another geographic region [37,39], included other physicians [30,31,34–36,38,40], or other sickness absentees [32,33,41]. Some of the interventions included 20 or fewer physicians in the intervention and control groups [32,33,38,39], had a high dropout rate (50% or more) [37], or a high contamination risk [32,33,39,40].

### Sickness absence and return to work

Several outcomes were reported in the nine intervention studies: SA, RTW, absenteeism, days off from work, and work resumption (Table 1). In all, 30 different types of effect measures were used in the 12 publications, that is, several publications used more than one measure (Table 1). There were also variations in how the outcomes were operationalized, examples are: duration of SA [34,35,37], lasting full RTW [30,31,38], time from first day of SA to first day of RTW [32,33,36,39–41], or as risk estimates for SA/RTW [30–41]. *Time to RTW* was calculated for actual days on SA before RTW [30,31,34–37], days since onset of SA to RTW [38], total hours on SA before RTW [36], number of workers with full RTW calculated from the first day of SA to the first day of full RTW [36], work resumption rate [39], or days to first, part-time, or full RTW [32,33,36,40,41]. The *risk estimates* were based on duration or number of workers who returned to work [36], work resumption rate [39], or days from first day of SA to first day of RTW [30–33,37,38,40,41].

The *information sources* for these outcomes varied, they were either self-reported [30,31,38], extracted from medical records [32,33,36,40], or obtained from administrative registers [32–37,39,41].

### The strategies and the content of the interventions

In the studies of most of the included interventions, there was no thorough description of the composition nor of the exact content of the intervention. The interventions described involved case-management principles, taking the time constraints of the physicians into account [30,31], establishing a-one-year postgraduate communication skills training course [36], using training based on a model previously developed for the primary healthcare setting [38] or being an assessed by a psychiatrist [38]. Furthermore, several interventions focused on how implementation of some type of guidelines impacted SA/RTW [34–39].

Two of the interventions were “complex” [40,41], in terms of directed towards both physicians and patients, and seven were “simple” interventions [30,32–39], that is, directed only to physicians – this does not necessarily mean that they were less comprehensive. All interventions included some training elements of varying duration for the physicians; a couple of hours [34,35,37], a five-days training [30–33], or a one-year training [36] (Table 1). One intervention also had additional follow-up meetings [30,31]. The content of the trainings were more or less comprehensive, ranging from a one-year postgraduate communication skills course [36] or combinations of several pedagogical approaches, to a one-day workshop including teamwork and role-playing [34,35]. In half of the intervention studies, the content of the training was not described, e.g. regarding type of pedagogic approach, content of lectures, etc., or its duration in detail [32–35,37,39,40].

### Intervention effects regarding patient’s sickness absence and return to work

Statistically significant positive (i.e. in intended direction) intervention effects from specific interventions were observed in four of the nine interventions (Supplementary Appendix table 2); three conducted in the Netherlands [32,33,38,41] and one in Norway [34,35]. Two other interventions showed a negative intervention effect [37,40], i.e. prolonged RTW and three of the interventions showed no statistically significant intervention effect on SA/RTW [30,31,36,39].

**Figure 2.**
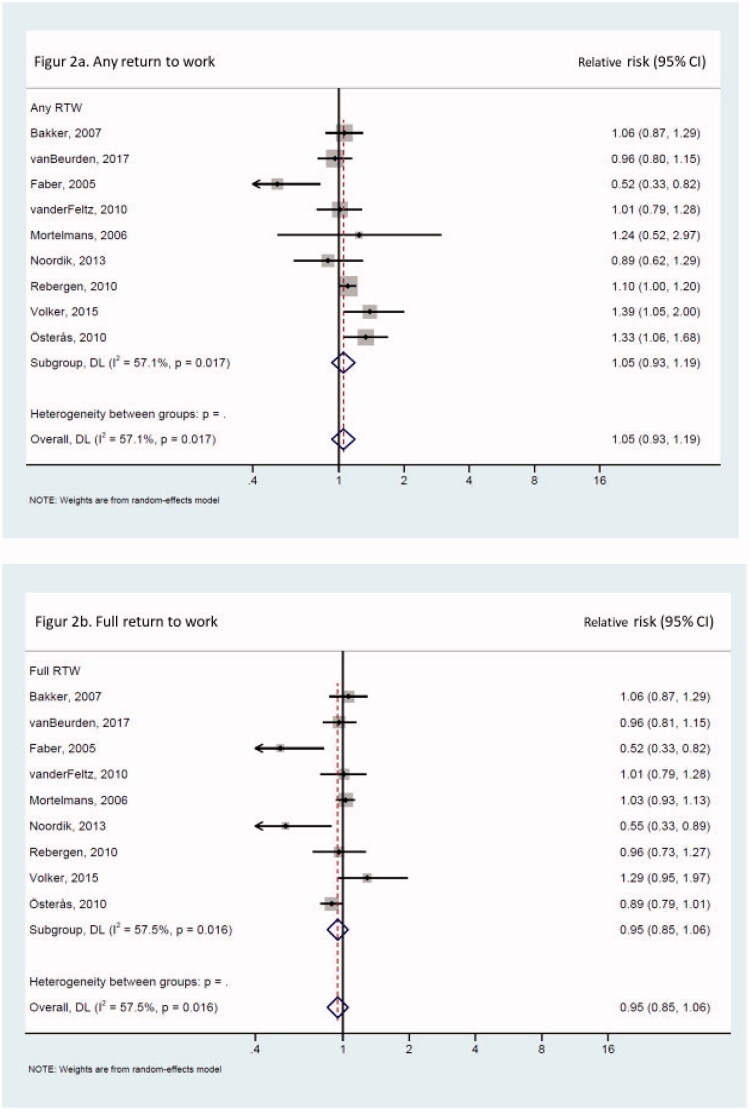
**(a**) Any return to work. **(**b) Full return to work.

In the included interventions, risk estimates with 95% CIs, or information required to calculate risk estimates, were presented, and these risk estimates were consequently included in two meta-analyses using a random-effect model (Figure 2). No statistically significant effect was observed in the main meta-analysis of any or full RTW, nor in meta-analyses stratified by type of intervention, design, or type of physicians targeted (Table 2). In analyses of geographical area, the two studies from the “rest of Europe” rendered a statistically significant result for any RTW (Table 2).

## Discussion

This systematic literature review summarized the interventions aimed at improving physicians’ sickness certification practice, with information on the physicians, intervention characteristics, as well as the intervention effects in terms of SA or RTW among their patients. The nine included intervention studies presented a wide range of intervention contents as well as many different effect measures for SA and RTW, which makes the comparison between the interventions challenging. No statistically significant effect in the meta-analyses in favor of the intervention was observed for *any RTW* (*first, partial, or full*), however, nor for *full RTW*.

Six additional controlled interventions studies with RTW/SA as effect measures were published after the previously two conducted reviews that included publications through October 2002 [10] and August 2009 [9], respectively. All included intervention studies were conducted in three European countries, no studies from other countries were observed in our search, or through contact with researchers in other countries. Although the European countries are similar in many ways, they have different social security systems. This might be one possible reason for the large variation in types of interventions and outcomes used, however, as the large variation also applies to interventions from the same country (the Netherlands) this does not appear to be the only explanation. It is noteworthy that as many as 30 different effect measures that relate to SA and RTW were used in the 12 publications. The problem with inconsistency in outcomes in this field is a problem that has been raised by many others [42–45], and there is clearly a need for developing joint measures regarding SA and RTW [46].

Only in four of the interventions, an intended intervention effect on SA or RTW was observed. One possible reason for this could be that *full RTW* was not an option given the studied patient's condition. SA and RTW are complex phenomena that incurs large costs to society, including costs for productivity loss, healthcare, and efforts to get the sickness absentee back to work, and can be affected by many other factors than the physicians’ competence and actions, e.g. changes in type and severity of disease or injury, multi-morbidity, sociodemographic factors, SA-insurance (rules and practices), employment frequency, age for old-age pension, labor-market factors, economic conditions [47–49], how sickness absentees experience encounters regarding work from different stakeholders [50–56], as well as different specific work-place factors [57–61]. Furthermore, the intention cannot be to simply shorten SA spells. Going back to work too soon might have negative consequences for both the patient’s colleagues and clients/costumers as well as for the future health and work capacity of the patient.

Two of the interventions were “complex”, i.e. the interventions had aspects also targeting the patients, which further complicated the assessment of whether the effect on the patient’s SA or RTW was related to the physicians’ sickness certification practice or to other components of the intervention. However, the other seven interventions were “simple” in terms of being directed only to the physicians, not to say they were less comprehensive since they were interventions based on changes in regulations or introductions of nationwide guidelines, and/or concerned collaboration with different stakeholders. SA or RTW as outcome measures might also depend on other factors such as guideline adherence.

Although no statistically significant improvement in RTW was observed in the overall meta-analysis, four individual studies favored the experimental intervention that aimed to improve sickness certification. Such improvement could be important at a patient, physician and societal level, warranting further research.

The outcomes SA or RTW were only measured for patients already on SA. Therefore, possible results regarding, e.g. if some individuals continued to work during their disease as a result of a changed sickness certification practice, were not observed. Also, the interventions varied regarding how long the studied patients had been on SA at inclusion. This ranged from the first day on SA [40] to having been on SA since up to three months [30,31,37].

Previously, it has been pointed out that there is a need for international comparisons of similar interventions in different countries, as a knowledge base for actions regarding physicians’ sickness certification practice [9,62]; this need is still not met.

### Strengths and limitations

The strength of this literature review with meta-analyses is the systematic approach regarding search of studies, and the wide scope to provide insight into the complex physician task of sickness certification. However, there were also limitations, the low number of controlled intervention studies, as well as the heterogeneity in intervention contents and outcome definitions. As with all systematic reviews, the risk of publication bias must be taken into consideration. We did not detect statistically significant indication of publication bias (by performing a funnel plot), however, due to the small number of included studies, we cannot dismiss it. In this literature review the extracted results were often reported together with other results, perhaps modifying the risk of only positive results being published. Further, the high proportion of RCTs which often have a protocol for which outcomes to report may also in some way limit the risk of publication bias. A quality appraisal of the studies was not an inclusion criterion, nor was there any intention of scientific grading of evidence. However, the quality of the included studies would probably be considered as predominantly low, e.g. many studies had small sample sizes, high number of dropouts (or this was not presented). In addition, some interventions were controlled or randomized on patient and not physician level [39,41]. Moreover, several studies lacked detailed descriptions of what components the intervention involved.

## Conclusion

The results from this systematic literature review with meta-analyses contribute to the body of knowledge by indicating that physicians’ sickness certification practice might be influenced by interventions, both in intended and in unintended directions. The results from the meta-analyses did not show a clear effect of the interventions in the intended direction regarding *any RTW* (*first, partial or full*), nor *full RTW* among patients. Given the heterogeneity in interventions and outcome measures, there is a great need for evaluation of different combinations of interventions, and consistent reporting on interventions strategies and on use of outcome measures.

## Supplementary Material

Supplemental Material

Supplemental Material

Supplemental Material

## Data Availability

All data relevant to the study are included in the article or uploaded as supplementary information. Data from 12 scientific publications have been used.

## Patient and public involvement

Patients and/or public were not involved in the design of this study
