# Supplementary material for: Interventions regarding physicians’ sickness certification practice – a systematic literature review with meta-analyses
Source: Scand J Prim Health Care. 2022 Mar 7;40(1):104–14. doi: 10.1080/02813432.2022.2036420 (PMC9090374; doi:10.1080/02813432.2022.2036420)
Supplement: Supplemental Material [file IPRI_A_2036420_SM2559.docx]

Appendix, table 1. Search log for publications concerning interventions regarding physicians’ sickness certification practice, with search terms formulated in accordance to PICO (Population, Intervention, Comparator, Outcome) framework.

| PICO | Search string | Database | Filter | Hits (n) |
| --- | --- | --- | --- | --- |
| Population | Search ("physician"[Title/Abstract]) OR "physician practice patterns"[Title/Abstract] | PubMed | Publication date from 2009/01/01 to 2018/03/09 | 62612 |
| Intervention | Search ((("education"[Title/Abstract]) OR "guidelines"[Title/Abstract]) OR "guidelines adherence"[Title/Abstract]) OR "intervention"[Title/Abstract] | PubMed | Publication date from 2009/01/01 to 2018/03/09 | 586299 |
| Outcome | Search (((((((("sick leave"[Title/Abstract]) OR "sickness certification"[Title/Abstract]) OR "medical certification"[Title/Abstract]) OR "sick listing"[Title/Abstract]) OR "disability evaluation"[Title/Abstract]) OR "disability insurance"[Title/Abstract]) OR "return to work"[Title/Abstract]) OR "work ability" | PubMed | Publication date from 2009/01/01 to 2018/03/09 | 7100 |
|  | Search ((((("physician"[Title/Abstract]) OR "physician practice patterns"[Title/Abstract]) AND ( "2009/01/01"[PDat] : "2018/03/09"[PDat] ))) AND ((((("education"[Title/Abstract]) OR "guidelines"[Title/Abstract]) OR "guidelines adherence"[Title/Abstract]) OR "intervention"[Title/Abstract]) AND ( "2009/01/01"[PDat] : "2018/03/09"[PDat] ))) AND (((((((((("sick leave"[Title/Abstract]) OR "sickness certification"[Title/Abstract]) OR "medical certification"[Title/Abstract]) OR "sick listing"[Title/Abstract]) OR "disability evaluation"[Title/Abstract]) OR "disability insurance"[Title/Abstract]) OR "return to work"[Title/Abstract]) OR "work ability"[Title/Abstract]) OR "work capacity evaluation"[Title/Abstract]) AND ( "2009/01/01"[PDat] : "2018/03/09"[PDat] ))  Search: (((("physicians"[Title/Abstract]) OR ("practice patterns physicians"[Title/Abstract]))) AND ((((("education"[Title/Abstract]) OR ("guidelines"[Title/Abstract])) OR ("guidelines adherence"[Title/Abstract])) OR ("assessment"[Title/Abstract])) OR (intervention[Title/Abstract]))) AND (((((((((((("sick leave"[Title/Abstract]) OR ("sickness certification"[Title/Abstract])) OR ("medical certification"[Title/Abstract])) OR ("medical certificate"[Title/Abstract])) OR ("sick listing"[Title/Abstract])) OR ("disability evaluation"[Title/Abstract])) OR ("disability insurance"[Title/Abstract])) OR ("return to work"[Title/Abstract])) OR ("work ability"[Title/Abstract])) OR ("work capacity evaluation"[Title/Abstract])) OR ("work inability"[Title/Abstract])) OR ("work incapacity"[Title/Abstract])) | PubMed | Publication date from 2009/01/01 to 2018/03/09  Update:  2018/03/10 to 2019/01/28  Update with added search terms:  2009/01/01 to 2020/06/15 | 629  50  117 |
| Population | **TOPIC:** (physician or "physicians practice patterns") | Web of Science | Indexes=SCI-EXPANDED, SSCI, A&HCI, CPCI-S, CPCI-SSH, ESCI. Timespan=2009-2018. | 138787 |
| Intervention | **TOPIC:** (education or guideline or "guideline adherence" or assessment or intervention) | Web of Science | Indexes=SCI-EXPANDED, SSCI, A&HCI, CPCI-S, CPCI-SSH, ESCI. Timespan=2009-2018. | 1837086 |
| Outcome | **TOPIC:** ("sick leave" or "sickness certification" or medical certification or "sick listing" or "disability evaluation" or "disability insurance" or "return to work" or "work ability" or "work capacity evaluation") | Web of Science | Indexes=SCI-EXPANDED, SSCI, A&HCI, CPCI-S, CPCI-SSH, ESCI. Timespan=2009-2018. | 9770 |
|  | **TOPIC:** (physician or "physicians practice patterns") AND **TOPIC:** (education or guideline or "guideline adherence" or assessment or intervention) AND **TOPIC:** ("sick leave" or "sickness certification" or medical certification or "sick listing" or "disability evaluation" or "disability insurance" or "return to work" or "work ability" or "work capacity evaluation")  TS=(physician or "physicians practice patterns") AND TS=(education or guideline or "guideline adherence" or assessment or intervention) AND TS=("sick leave" or "sickness certification" or medical certification or "sick listing" or "disability evaluation" or "disability insurance" or "return to work" or "work ability" or "work capacity evaluation") AND TS=("work inability" or "work incapacity" or "work capacity" or "medical certificate" or "insurance medicine")) AND LANGUAGE: (English)  Indexes=SCI-EXPANDED, SSCI, A&HCI, CPCI-S, CPCI-SSH, ESCI Timespan=2009-2020 | Web of Science | Indexes=SCI-EXPANDED, SSCI, A&HCI, CPCI-S, CPCI-SSH, ESCI. Timespan=2009-2018.  Update with added search terms:  2020/06/15 | 643  105  150 |

Appendix, table 2. Characteristics of the nine included intervention (12 publications) regarding physicians’ sickness certification practice.

| -First author  -Publication year  -Country | Aim | a) Study design  b) Setting  c) Year of inclusion  d) Duration of follow-up | a) Participating/ Eligible physicians  b) Dropout  c) %♀ | a) Sickness absent patients  b) Dropout  c) %♀ | a) Type of intervention  b) Intervention components  c) Also directed at patients (“complex” intervention) | a) Type of data  b) Method for analyses  c) Outcome measures | a) Results  b) Direction of intervention effect  c) Estimates of effect sizes | Comments |
| --- | --- | --- | --- | --- | --- | --- | --- | --- |
| -Bakker  -1)2007  -2)2010  -Netherlands  (23, 24) | 1)To assess the effectiveness of our Minimal Intervention for Stress-related mental disorders with SA (MISS) in primary care, which is intended to reduce SA and prevent chronicity of symptoms.  2) To study the effects of a brief patient-stress management training on the performance of  general practitioners (GPs). | a) RCT, randomised on physician level b) Primary healthcare practice c) Sept? 2003-Oct? 2004  d) 1 year, not clear from the publication | a) 46/139 GPs  IG=24  CG=22  b) IG 95.8%  CG 100%  c) 50/50%♀ | a) 306 patients on SA since <3 months due to stress-related mental disorders,  IG=167 of 227  CG=139 of 206  b) 73.6%/67.5%  c) baseline 67%/65% ♀ | a) Training  b) Program of 11 h, 2 sessions with 2 follow ups, during a period of 6-10 weeks, GP skills course map used for the training, training with 10 min consultations for patients  c) No | a) SA data self-reported  b) Cox regression  c) 1) Duration of SA until ‘lasting full RTW’ | a) No significant difference on duration of SA until full RTW between groups regardless of MISS education.  b) No effect  c) HR 1.06; 95% CI 0.87–1.29 (used in meta-analysis concerning both some and full RTW) | Also, clinical outcomes among the patients were measured |
| -van Beurden  -2017  -Netherlands  (29) | To evaluate whether this intervention leads to earlier RTW in workers with CMD. | a) RCT, randomised on physician level b) Occupational health service  c) OPs Oct 2010 to Jan 2011, workers Jan 2012 to Feb 2014  d) 2 years | a) 65/155 OPs IG=31, CG=34  b) IG 78.1%, CG 79.4%  c) 34.6%/18.6% ♀ | a) 3379 workers on SA due to CMD IG=1493, CG=1886  b) 95.7/95.4%  c) 60.5%/56.7% ♀ | a) Training  b) Postgraduate communication skills training course, 1 year resulting in educational credits  c) No | a) SA data from occupational health service  b) Kaplan–Meier survival analysis, Cox regression  c) Full RTW, days to full RTW, first RTW, days to first RTW, total number of SA hours | a) No significant differences for first RTW between groups nor on days to full RTW (working  the number of hours of their employment contract, for at  least 4 weeks was considered a full RTW) regardless of communication skills.  b) No effect  c) HR full RTW 0.96; 95% CI 0.81–1.15, first RTW 0.96; 95% CI 0.80–1.15 |  |
| -Faber  -2005  -Netherlands  (30) | To determine the effectiveness of a training to increase collaboration between general practitioners and occupational health physicians in the treatment of patients with low back pain because more collaboration might improve a patient’s recovery and shorten SA. | a) CT, controlled on region level before/after  b) Primary healthcare practice and occupational health service c) Year of inclusion not presented  d) 6 months | a) 49 GPs, 47 OPs of all GPs and OPs in two regions  Intervention region 21 GPs, 20 OP Control region 28 GPs, 27 OPs  b) First follow-up GPs 52%, OPs 75%; last follow-up GPs 25%, OPs 45%  c) ? % ♀ | a) 112 patients on SA 3-12 weeks due to back pain  Intervention region 56  Control region 56  b) 100%  c) 29%/21% ♀ | a) Training, guideline  b) A protocol, learn to work together, 4-5 h, protocol - policy according to guidelines, suggested moments and context of collaboration, collaboration was recommended after 6 weeks of low back pain  c) No | a) SA data from occupational health service  b) Kaplan-Meier survival curve  c) Duration of SA | a) Significant, not intended, prolonged RTW for IG when used a collaboration protocol.  b) Unfavourable effect  c) HR=0.52 p=0.005, 95% CI 0.33–0.82 (CI calculated using the presented p-value, used in meta-analysis concerning both some and full RTW) | Also, outcomes regarding pain, functional disability, quality of life, satisfaction with GP, and medical consumption among the patients were measured.  CI was not reported for analysis of HR, and information required to calculate CI was not presented. |
| -van der Feltz Cornelis  -2010  -Netherlands  (31) | To assess time to RTW after a psychiatric consultation providing treatment advice to the OP for employees on SA with CMD in the occupational health setting, compared to care as usual. | a) RCT, randomised on physician level  b) Occupational health service  c) Year of inclusion not presented d) 6 months | a) 24/64 OPs IG=12, CG=12  b) ?  c) ? % ♀ | a) 60 patients on SA ≥ 6 weeks due to CMD  IG=29  CG=31  b) 86.2/77.4%  c) 64% /52%♀ | a) Training, guideline  b) Training in diagnosis and treatment and psychiatric consultation, with stepped care protocol supported by a liaison-consultation function, presentation of the stepped care protocol three-monthly regular meetings for continuing education of caregivers  c) No | a) SA data self-reported  b) logistic regression, multi-level analysis, Kaplan-Meier survival  analysis, Cox regression  c) Time to (lasting) RTW | a) Significant difference on time to full RTW without relapse at 3 months follow-up with stepped care protocol compared to care as usual in absolute but not in relative numbers (non-significant at 6 months follow-up).  b) Partly intended effect  c) RR full RTW at 6 months 1.0, 95% CI 0.79-1.28 (RR and CI calculated from reported number of patients who no longer were on SA or who had returned to work, in both the IG and the CG, used in meta-analysis concerning both some and full RTW) | Also, clinical outcomes among the patients were measured. Risk estimates not presented in the article, RR was measured from number of individuals with the outcomes in the IG and the CG. |
| -Mortelmans  -2006  -Belgium  (32) | To assess the influence of enhanced information exchange between physicians on patient outcome. | a) CT, controlled on patient level, a control region  b) Occupational health service  c) 2001 to 2002  d) One year from SA | a) All 15 social insurance physicians and 40 OPs at the centers  b) ?  c) ? % ♀ | a) 1564 patients on SA <1-month IG:505  CG: 1059  b) 84% among all patients  c) 59%/46%♀ | a) Training, checklist  b) Communication form, education - case studies of patients with extended SA duration, discussions; physicians’ job tasks and responsibilities, their perception of each other, collaboration experiences and problems  c) No | a) SA data from occupational health service  b) t-test, chi-square, Fisher’s exact test, Mann-Whitney U test, Kaplan-Meier survival analysis, logistic regression  c) Gradual and full work resumption rate calculated from non-dependent of benefits | a) No significant difference on work resumption rate regardless of communication form between stakeholders.  b) No effect  c) RR full RTW 1.03, 95% CI 0.93–1.13, gradual RTW 1.24, 95% CI 0.52-2.97 | Also, status for SA benefit dependency among the patients was measured. |
| -Noordik  -2013  -Netherlands  (33) | To evaluate the effectiveness of the RTW-E intervention, applied by OPs, in reducing the time-to-full RTW of workers on SA due to CMD in comparison to care as usual. | a) RCT, randomised on physician level  b) Occupational health service  c) Nov 2006 to Dec 2007  d) 12 months | a) 56/74 OPs  IG=28  CG=28  b) 62.5%  c) ? % ♀ | a) 160 workers at the start of their SA IG=75  CG= 85  b) T1 83.6/94.0%  T2 78.1/81.0%  T3 74.0/73.8%  T4 69.9/77.4%  c) 75.7%/66.7% ♀ | a) Training, IT support  b) 2-days training in the RTW-E program. 3 follow-up tutorial sessions during the inclusion period, discussions; difficulties and practical solutions with supervisor and other participating OP, CAU and gradually exposed in vivo to more demanding work situations structured by a hierarchy of tasks evoking increasing levels of anxiety, stress, or anger  c) Yes | a) SA data from medical records  b) Kaplan-Meier survival  analysis, Cox regression, Wald test, linear mixed model  c) Time to full RTW ≥28 calendar days without a recurrence of SA time to partial RTW, the number of recurrences of SA | a) Workers on SA due to CMD treated with RTW-E had a prolonged time-to-full. RTW  b) Unfavourable effect  c) HR full RTW 0.55, 95% CI 0.33–0.89, partial RTW 0.89, 95% CI 0.62-1.29 | Also clinical outcomes and satisfaction with OP among the patients were measured. |
| -Rebergen  -1) 2009  -2) 2010  -Netherlands  (25, 26) | 1)To evaluate the effectiveness of guideline-based care of workers with mental health disorders, which promotes counselling by the OP facilitating RTW.  2)To examine the (adherence of OP) to the Dutch guideline on the management of CMDs and its) effect on RTW as part of the process evaluation of a trial comparing adherence to the guideline to care as usual. | a) RCT, randomised on worker level b) Occupational health service c) 2002 to 2005  d) One year | a) All 5 OPs  b) ?  c) 60% ♀ | a) 240 workers on SA due to mental health disorders IG=125  CG=115  b) 100%  c) 48.8/49.5%% ♀ | a) Training, guideline  b) Guideline-based care, 3-day training, Dutch national guideline on OP management of workers with CMDs, encouraged to use specific tools, such as symptom questionnaires, patient information leaflets on stress, and day structuring exercises.  c) No | a) SA data from workplace register, medical records  b) t-test, chi-square, Kaplan-Meier survival analysis, Cox regression, linear regression  c) Time to full RTW, partial RTW, duration partial RTW, time to full RTW median in days | a) Significant difference for partial RTW before full RTW, and a significant association between performance indicator for guideline adherence, and shorter time to first and full RTW  b) Partly intended effect  c) HR full RTW 1.1, 95% CI 1.0–1.2, first RTW 1.1, 95% CI 1.0–1.2 | Also, productivity loss and treatment satisfaction among the patients were measured, and guideline adherence among the physicians. |
| -Volker  -2015  -Netherlands  (34) | To evaluate the effects of the ECO intervention on time to RTW and mental health outcomes. | a) RCT, randomised on patient level  b) Occupational health service  c) Jul 2011 to Jan 2013  d) 1.5 years | a) All 60 OPs IG=31, CG=29  b) ?  c) ? % ♀ | a) 220 employees on SA 4-26 weeks due to CMD  IG=131  CG=89  b) T1 70.2%/74.2%  T2 67.2%/78.7%  T3 55.7%/71.9%  T4 56.5%/64.0%  c) 58.8%/60.0% ♀ | a) Training, guideline  b) Structured functional assessment, 1-day workshop  c) Yes | a) SA data from occupational health service and employer  b) t-tests, Kaplan-Meier survival analysis, Cox regression  c) Time to first partial or full RTW, time to full RTW | a) Significant difference on first RTW but not on full RTW with structured functional assessment  b) Partly intended effect  c) HR full RTW 1.29, 95% CI 0.95-1.97; first RTW 1.39, 95% CI 1.05-2.00. | Also, clinical outcomes among the patients were measured. |
| -Østerås  -2009  -2010  -Norway  (27, 28) | 1) To evaluate  intervention effects on important GP parameters; knowledge, attitudes, self-efficacy towards functional  assessments and knowledge about patient work factors  2)To analyse intervention effects on GPs’  sick-listing practice and patient SA. | a) RCT, randomised on physician level  b) Primary healthcare practice  c) 1 March to 31 October 2005  d) 6 months | a) 57/360 GPs IG=28, CG=29  b) T1 78.6/93.1%  T2 78.6/ 89.7%  c) 34.8%/38% ♀ | a) 2170 SA spells during the intervention period  CG=1231  IG=939  b) 100%  c) ? | a) Training  b) Structured method in general practice for assessing functional ability in patients with long-term SA, workshop including teamwork and role-playing, 1-day workshop  c) No | a) SA data from register  b) Cox regression, two level regression  c) Duration of patient SA episodes (mean number of days), part-time SA, active SA (enables people on SA to attend work doing other tasks than they normally do) | a) Significant difference for more part-time SA, and for less individuals on active SA but not for full RTW with structured method in assessing functional ability  b) Partly intended effect  c) HR full RTW 0.89; CI 0.79-1.01, OR part-time SA 1.33, CI 1.06-1.68, HR less individuals on active SA 0.65, CI 0.43-0.98 | Also, vocational rehabilitation among the patients was measured. |

NOTE GP=general practitioner, OP=occupational physician, CMD=common mental disorder, RCT=randomised controlled trial, CT=controlled trial, SA=sickness absence, RTW=return to work, MISS=Minimal Intervention for Stress-related mental disorders with SA, IG= intervention group, CG=control group, HR=hazard ratio, OR=odds ratio, RR=relative risk, CI=confidence interval, n/a=not applicable

**Appendix 3**

**Publications excluded after full-text screening**

Arrelöv B, Borgquist L, Ljungberg D, Svärdsudd K. The influence of change of legislation concerning sickness absence on physicians’ performance as certifiers: A population-based study. Health policy. 2003;63(3):259-68. doi:10.1016/S0168-8510(02)00081-7.

Bremander AB, Hubertsson J, Petersson IF, Grahn B. Education and Benchmarking Among Physicians May Facilitate Sick-Listing Practice. Journal of Occupational Rehabilitation.22(1):78-87.

Buchbinder R, Jolley D. Improvements in general practitioner beliefs and stated management of back pain persist 4.5 years after the cessation of a public health media campaign. Spine. 2007;32(5):56-62.

Carlsen B, Frithjof Norheim O. Introduction of the patient-list system in general practice Changes in Norwegian physicians’ perception of their gatekeeper role. Scandinavian Journal of Primary Health Care. 2003;21(4):209-13. doi:10.1080/02813430310004155.

Claussen B. Rehabilitation efforts before and after tightening eligibility for disability benefits in Norway. International journal of rehabilitation research Internationale Zeitschrift fur Rehabilitationsforschung Revue internationale de recherches de readaptation. 1997;20(2):139-47.

Claussen B. Physicians as gatekeepers: will they contribute to restrict disability benefits? Scandinavian Journal of Primary Health Care. 1999;16(4):199-203. doi:10.1080/028134398750002954.

Cohen D, Khan S, Allen J, Sparrow N. Shifting attitudes: the National Education Programme for work and health. Occup Med (Lond). 2012;62(5):371-4. doi:10.1093/occmed/kqs081.

Cohen D, Khan S, Marfell N. Fit for work? Evaluation of a workshop for rheumatology teams. Occupational medicine (Oxford, England). 2016;66(4):296-9.

Coole C, Watson PJ, Thomson L, Hampton R. How do GPs complete fit note comments? Occup Med (Lond). 2013;63(8):575-8. doi:10.1093/occmed/kqt126.

de Brouwer CPM, Verdonk P, van Amelsvoort L, Jansen NWH, Kant I, Widdershoven GAM. Experiences of occupational physicians with the implementation of indicated prevention for long term sickness absence. Work. 2017;57(2):157-72. doi:10.3233/wor-172547.

de Kock CA, Lucassen P, Bor H, Knottnerus JA, Buijs PC, Steenbeek R et al. Training GPs to improve their management of work-related problems: results of a cluster randomized controlled trial. Eur J Gen Pract. 2018;24(1):258-65. doi:10.1080/13814788.2018.1517153.

Dekkers-Sanchez PM, Wind H, Frings-Dresen MHW, Sluiter JK. Implementation of a checklist to assess factors relevant for work ability assessments of employees on long-term sick leave. International Archives of Occupational and Environmental Health. 2015;88(5):577-88.

Dey P, Simpson CW, Collins SI, Hodgson G, Dowrick CF, Simison AJ et al. Implementation of RCGP guidelines for acute low back pain: a cluster randomised controlled trial. The British journal of general practice: the journal of the Royal College of General Practitioners. 2004;54(498):33-7.

Englund L, Tibblin G, Svärdsudd K. Effects on physicians' sick-listing practice of an administrative reform narrowing sick-listing benefits. Scandinavian Journal of Primary Health Care. 2000;18(4):215-9. doi:10.1080/028134300448779.

Faber E, Bierma-Zeinstra SM, Burdorf A, Nauta AP, Hulshof CT, Overzier PM et al. In a controlled trial training general practitioners and occupational physicians to collaborate did not influence sickleave of patients with low back pain. J Clin Epidemiol. 2005;58(1):75-82. doi:10.1016/j.jclinepi.2004.04.015.

Foster NE, Mullis R, Hill JC, Lewis M, Whitehurst DG, Doyle C, Konstantinou K, Main C, Somerville S, Sowden G, Wathall S, Young J, Hay EM. Effect of stratified care for low back pain in family practice (IMPaCT Back): a prospective population-based sequential comparison. Annals of family medicine. 2014;12(2):102-11. doi:10.1370/afm.1625.

Gustavsson C, Hinas E, Ljungquist T, Alexanderson K. Obstetricians/Gynecologists' Problems in Sickness Certification Consultations: Two Nationwide Surveys. Obstet Gynecol Int. 2016:11. doi:10.1155/2016/9421316.

Gustavsson C, Hinas E, Ljungquist T, Alexanderson K. General practitioners' use of sickness certification guidelines in Sweden at introduction and four years later: a survey study. International journal for quality in health care: journal of the International Society for Quality in Health Care. 2018;30(6):429-36. doi:10.1093/intqhc/mzy044.

Hussey L, Money A, Gittins M, Agius R. Has the fit note reduced general practice sickness certification rates? 2015;65(3):182-9.

Joosen MC, van Beurden KM, Terluin B, van Weeghel J, Brouwers EP, van der Klink JJ. Improving occupational physicians’ adherence to a practice guideline: feasibility and impact of a tailored implementation strategy. BMC Medical Education. 2015;15(1):82. doi:10.1186/s12909-015-0364-8.

Kersnik J. Management of sickness absence: a quality improvement study from Slovenia. Quality in Health Care. 1999;8(4):262. doi:10.1136/qshc.8.4.262.

Kiessling A, Arrelöv B. Sickness certification as a complex professional and collaborative activity - a qualitative study. BMC Public Health. 2012;12(1):702. doi:10.1186/1471-2458-12-702.

Kok R, Hoving JL, Smits PBA, Ketelaar SM, van Dijk FJH, Verbeek JH. A Clinically Integrated Post-Graduate Training Programme in Evidence-Based Medicine versus 'No Intervention' for Improving Disability Evaluations: A Cluster Randomised Clinical Trial. Plos One. 2013;8(3):9.

Lambeek LC, van Mechelen W, Buijs PC, Loisel P, Anema JR. An integrated care program to prevent work disability due to chronic low back pain: a process evaluation within a randomized controlled trial. 2009;10:147.

Lofgren A, Silen C, Alexanderson K. How physicians have learned to handle sickness-certification cases. Scand J Public Health. 2011;39(3):245-54. doi:10.1177/1403494810393301.

Maiwald K, Meershoek A, de Rijk A, Nijhuis FJN. Policy on professional support in return-to-work: Occupational health professionals' experiences in a Canadian setting. Work (Reading, Mass). 2015;53(1):143. doi:10.3233/WOR-152141.

Malcolm RM, Harrison J, Forster H. Effects of changing the pattern of sickness absence referrals in a local authority. Occupational medicine (Oxford, England). 1993;43(4):211. doi:10.1093/occmed/43.4.211.

Martijn MR, Marie-Christine JP, Monique HWF-D, Judith KS. Feasibility and acceptability of a workers’ health surveillance program for hospital physicians. International journal of occupational medicine and environmental health. 2015;28(4):731-9. doi:10.13075/ijomeh.1896.00420.

Meeuwissen JAC, van der Feltz-Cornelis CM, van Marwijk HWJ, Rijnders PBM, Donker MCH. A stepped care programme for depression management: an uncontrolled pre-post study in primary and secondary care in The Netherlands. International Journal of Integrated Care. 2008;8:e05.

Money A, Hann M, Turner S, Hussey L, Agius R. The influence of prior training on GPs' attitudes to sickness absence certification post-fit note. 2015;16(5):528-39.

Mortelmans AK, Donceel P, Lahaye D, Bulterys S. An analysis of the communication during an enhanced and structured information exchange between social insurance physicians and occupational physicians in disability management in Belgium. Disabil Rehabil. 2007;29(13):1011-20. doi:10.1080/09638280600929003.

Mortelmans K, Donceel P, Lahaye D. Disability management through positive intervention in stakeholders information asymmetry. A pilot study. Occupational Medicine. 2006;56(2):129-36. doi:10.1093/occmed/kqj014.

Nilsing E, Soderberg E, Oberg B. Sickness certificates in Sweden: did the new guidelines improve their quality? Bmc Public Health. 2012;12:9. doi:10.1186/1471-2458-12-907.

Nordhagen HP, Harvey SB, Rosvold EO, Bruusgaard D, Blonk R, Mykletun A. Case-specific colleague guidance for general practitioners' management of sickness absence. Occup Med (Lond). 2017;67(8):644-7. doi:10.1093/occmed/kqx120.

Peppers D, Figoni SF, Carroll BW, Chen MM, Song S, Mathiyakom W. Influence of Functional Capacity Evaluation on Physician's Assessment of Physical Capacity of Veterans With Chronic Pain: A Retrospective Analysis. PM & R : the journal of injury, function, and rehabilitation. 2017;9(7):652-9. doi:10.1016/j.pmrj.2016.10.011.

Ratzon NZ, Amit Y, Friedman S, Zamir S, Rand D. Functional capacity evaluation: does it change the determination of the degree of work disability? Disability and health journal. 2015;8(1):80-5. doi:10.1016/j.dhjo.2014.08.004.

Rinsky‐Halivni L, Lerman Y. Discussion group networks in occupational medicine: A tool for continuing education to promote the integration of workers with disabilities. American Journal of Industrial Medicine. 2018;61(4):344-50. doi:10.1002/ajim.22818.

Sallis A, Birkin R, Munir F. Working towards a 'fit note': an experimental vignette survey of GPs. British Journal of General Practice.60(573):245-50.

Scheel IB, Hagen KB, Herrin J, Oxman AD. A randomized controlled trial of two strategies to implement active sick leave for patients with low back pain. Spine. 2002;27(6):561-6.

Scheel IB, Hagen KB, Oxman AD. Active sick leave for patients with back pain: all the players onside, but still no action. Spine. 2002;27(6):654-9.

Schellart AJ, Zwerver F, Anema JR, Van der Beek AJ. The influence of applying insurance medicine guidelines for depression on disability assessments. BMC research notes. 2013;6:225. doi:10.1186/1756-0500-6-225.

Schwarze M, Spallek M, Korallus C, Manecke IA, Teumer F, Wrbitzky R et al. Advantages of the JobReha discharge letter: an instrument for improving the communication interface in occupational rehabilitation. Int Arch Occup Environ Health. 2013;86(6):699-708. doi:10.1007/s00420-012-0805-1.

Sehlbach C, Govaerts MJB, Mitchell S, Rohde GGU, Smeenk F, Driessen EW. Box-ticking and Olympic high jumping - Physicians' perceptions and acceptance of national physician validation systems. Med Teach. 2018;40(9):886-91. doi:10.1080/0142159x.2018.1470320.

Skaner Y, Nilsson GH, Arrelov B, Lindholm C, Hinas E, Wilteus AL et al. Use and usefulness of guidelines for sickness certification: results from a national survey of all general practitioners in Sweden. Bmj Open. 2011;1(2):9.

Slebus FG, Kuijer PFM, Willems J, Frings-Dresen MHW, Sluiter JK. Work ability assessment in prolonged depressive illness. Occupational Medicine-Oxford.60(4):307-9.

Spanjer J, Krol B, Brouwer S, Popping R, Groothoff JW, van der Klink JJL. Reliability and Validity of the Disability Assessment Structured Interview (DASI): A Tool for Assessing Functional Limitations in Claimants. Journal of Occupational Rehabilitation. 2010;20(1):33-40.

Spanjer J, van de Mei S, Cornelius B, Brouwer S, van der Klink J. Effects of a training in the Disability Assessment Structured Interview on the interviews of Dutch insurance physicians. Disability and Rehabilitation. 2016;38(16):1632-41.

Strahl A, Gerlich C, Alpers GW, Ehrmann K, Gehrke J, Muller-Garnn A et al. Development and evaluation of a standardized peer-training in the context of peer review for quality assurance in work capacity evaluation. BMC Med Educ. 2018;18(1):135. doi:10.1186/s12909-018-1233-z.

Tamminga SJ, Verbeek J, Bos M, Fons G, Kitzen J, Plaisier PW, et al. Effectiveness of a Hospital-Based Work Support Intervention for Female Cancer Patients - A Multi-Centre Randomised Controlled Trial. Plos One. 2013;8(5):9.

van Beurden KM, Brouwers EPM, Joosen MCW, de Boer MR, van Weeghel J, Terluin B et al. Effectiveness of an Intervention to Enhance Occupational Physicians' Guideline Adherence on Sickness Absence Duration in Workers with Common Mental Disorders: A Cluster-Randomized Controlled Trial. Journal of Occupational Rehabilitation. 2017;27(4):559-67.

van Beurden KM, van der Klink JJL, Brouwers EPM, Joosen MCW, Mathijssen JJP, Terluin B et al. Effect of an intervention to enhance guideline adherence of occupational physicians on return-to-work self-efficacy in workers sick-listed with common mental disorders. Bmc Public Health. 2015;15:10.

van Dijk P, Hogervorst W, Riet Gt, van Dijk F. A protocol improves GP recording of long-term sickness absence risk factors. Occupational Medicine. 2008;58(4):257-62. doi:10.1093/occmed/kqn017.

van Rijssen HJ, Schellart AJM, Anema JR, de Boer WEL, van der Beek AJ. Systematic development of a communication skills training course for physicians performing work disability assessments: from evidence to practice. BMC Medical Education. 2011;11(1):28-. doi:10.1186/1472-6920-11-28.

van Rijssen J, Schellart AJM, Anema JR, van der Beek AJ. Communication skills training for physicians performing work disability assessments increases knowledge and self-efficacy: results of a randomised controlled trial. Disability and rehabilitation. 2015:1-9.

van Staa A, Burdorf A, Faber E, Miedema Harald S, Verhaar Jan A. Qualitative evaluation of a form for standardized information exchange between orthopedic surgeons and occupational physicians. BMC Health Services Research. 2006;6(1):144. doi:10.1186/1472-6963-6-144.

van Velzen JM, van Bennekom CAM, van Dormolen M, Sluiter JK, Frings-Dresen MHW. Evaluation of the implementation of the protocol of an early vocational rehabilitation intervention for people with acquired brain injury. Disability and Rehabilitation. 2016;38(1):62-70. doi:10.3109/09638288.2015.1017057.

Vandergrift JL, Gray BM, Weng W. Do State Continuing Medical Education Requirements for Physicians Improve Clinical Knowledge? Health Serv Res. 2018;53(3):1682-701. doi:10.1111/1475-6773.12697.

Wind H, Gouttebarge V, Kuijer P, Sluiter JK, Frings-Dresen MHW. Complementary value of functional capacity evaluation for physicians in assessing the physical work ability of workers with musculoskeletal disorders. International Archives of Occupational and Environmental Health. 2009;82(4):435-43.

Wind H, Gouttebarge V, Kuijer PP, Sluiter JK, Frings-Dresen MH. Effect of Functional Capacity Evaluation information on the judgment of physicians about physical work ability in the context of disability claims. Int Arch Occup Environ Health. 2009;82(9):1087-96. doi:10.1007/s00420-009-0423-8.

Volker D, Zijlstra-Vlasveld MC, Brouwers EPM, van der Feltz-Cornelis CM. Process Evaluation of a Blended Web-Based Intervention on Return to Work for Sick-Listed Employees with Common Mental Health Problems in the Occupational Health Setting. Journal of Occupational Rehabilitation. 2017;27(2):186-94. doi:10.1007/s10926-016-9643-4.

Zwerver F, Schellart AJ, Knol DL, Anema JR, van der Beek AJ. An implementation strategy to improve the guideline adherence of insurance physicians: an experiment in a controlled setting. Implementation Science. 2011;6(1):131. doi:10.1186/1748-5908-6-131.
