# Supplementary material for: Interventions regarding physicians’ sickness certification practice – a systematic literature review with meta-analyses
Source: Scand J Prim Health Care. 2022 Mar 7;40(1):104–14. doi: 10.1080/02813432.2022.2036420 (PMC9090374; doi:10.1080/02813432.2022.2036420)
Supplement: Supplemental Material [file IPRI_A_2036420_SM2553.pdf]

Systematic review

1. * Review title.

Give the title of the review in English

Reviews of interventions on physicians' sickness certification

2. Original language title.

For reviews in languages other than English, give the title in the original language. This will be displayed with the English language title.

3. * Anticipated or actual start date.

Give the date the systematic review started or is expected to start.

01/03/2018

4. * Anticipated completion date.

Give the date by which the review is expected to be completed.

31/05/2020

5. * Stage of review at time of this submission.

Tick the boxes to show which review tasks have been started and which have been completed. Update this field each time any amendments are made to a published record.
Reviews that have started data extraction (at the time of initial submission) are not eligible for inclusion in PROSPERO. If there is later evidence that incorrect status and/or completion date has been supplied, the published PROSPERO record will be marked as retracted.
This field uses answers to initial screening questions. It cannot be edited until after registration.

The review has not yet started: No

**Review stage**	**Started**	**Completed**
Preliminary searches	Yes	No
Piloting of the study selection process	Yes	No
Formal screening of search results against eligibility criteria	Yes	No
Data extraction	Yes	No
Risk of bias (quality) assessment	No	No
Data analysis	Yes	No

Provide any other relevant information about the stage of the review here.

The identified publications were only assessed for relevance according to the inclusion criteria, as degree of

quality was not an inclusion criterion in this study.

The identified publications were only assessed for relevance according to the inclusion criteria, as degree of quality was not an inclusion criterion in this study.

6. * Named contact.

The named contact is the guarantor for the accuracy of the information in the register record. This may be any member of the review team.

Mirkka Söderman

Email salutation (e.g. "Dr Smith" or "Joanne") for correspondence:

Mrs Söderman

7. * Named contact email.

Give the electronic email address of the named contact.

mirkka.soderman@ki.se

8. Named contact address

Give the full institutional/organisational postal address for the named contact.

Division of Insurance Medicine, Department of Clinical Neuroscience

Karolinska Institutet

SE-171 77 Stockholm, Sweden

9. Named contact phone number.

Give the telephone number for the named contact, including international dialling code.

08-524 832 24 (the administrator)

10. * Organisational affiliation of the review.

Full title of the organisational affiliations for this review and website address if available. This field may be completed as 'None' if the review is not affiliated to any organisation.

Division of Insurance Medicine, Department of Clinical Neuroscience, Karolinska Institutet

Organisation web address:

ki.se/im

11. * Review team members and their organisational affiliations.

Give the personal details and the organisational affiliations of each member of the review team. Affiliation refers to groups or organisations to which review team members belong. **NOTE: email and country now MUST be entered for each person, unless you are amending a published record.**

Mrs Mirkka Söderman. Karolinska Institutet, Mälardalen University
Assistant/Associate Professor Agneta Wennman-Larsen. Karolinska Institutet, Sophiahemmet University
Assistant/Associate Professor J.L. Hoving. Akademisch Medisch Centrum Universiteit van Amsterderdam
Professor Kristina Alexanderson. Karolinska Institutet

Dr Emilie Friberg. Karolinska Institutet

12. * Funding sources/sponsors.

Details of the individuals, organizations, groups, companies or other legal entities who have funded or sponsored the review.

Doctoral School in Health Care Sciences at Karolinska Institutet

Grant number(s)

State the funder, grant or award number and the date of award

13. * Conflicts of interest.

List actual or perceived conflicts of interest (financial or academic).

None

14. Collaborators.

Give the name and affiliation of any individuals or organisations who are working on the review but who are not listed as review team members. **NOTE: email and country must be completed for each person, unless you are amending a published record.**

15. * Review question.

State the review question(s) clearly and precisely. It may be appropriate to break very broad questions down into a series of related more specific questions. Questions may be framed or refined using PI(E)COS or similar where relevant.

The aim of these reviews is to obtain more knowledge about contents and effects of published interventions on physicians' sickness certification competence and/or practices as well as on patient's sickness absence and return to work.

Research question

Can physicians' sickness certification competence and/or practices be influenced through interventions?

16. * Searches.

State the sources that will be searched (e.g. Medline). Give the search dates, and any restrictions (e.g. language or publication date). Do NOT enter the full search strategy (it may be provided as a link or attachment below.)

Both the specific review, publications will be searched in two databases: PubMed and Web of Science. [illegible] context relating to the topic, and the spread of information available will be assessed by using these citation databases, and a search string including actual fields will be applied. In line with recommendations, the search will only include peer-reviewed journals.

Once relevant publications have been identified, further electronic tracking of citations, references, and of the author names found in these publications will be undertaken.

In addition, communication with other researchers active within this area will be made to identify further

potentially relevant studies for inclusion not found in the search.

Restrictions:

Peer-reviewed publications in the English language covering the years 2009-2020.

Intervention studies included in previous systematic literature reviews on physicians' sickness certification competence and/or practice, covering literature from before November 2002 and from January 2002 to August 2009, will also be included to capture all potential relevant studies.

Additional search strategy information can be found in the attached PDF document (link provided below).

17. URL to search strategy.

Upload a file with your search strategy, or an example of a search strategy for a specific database, (including the keywords) in pdf or word format. In doing so you are consenting to the file being made publicly accessible. Or provide a URL or link to the strategy. Do NOT provide links to your search **results**.

https://www.crd.york.ac.uk/PROSPEROFILES/119697_STRATEGY_20181217.pdf

Alternatively, upload your search strategy to CRD in pdf format. Please note that by doing so you are consenting to the file being made publicly accessible.

Do not make this file publicly available until the review is complete

18. * Condition or domain being studied.

Give a short description of the disease, condition or healthcare domain being studied in your systematic review.

Sickness certification is one of a physicians' medical tasks, and includes the complexity of the clinical assessment.

Physicians' sickness certification praxis includes tasks such as: to determine whether there is a disease or injury, and whether that results in a reduced ability to work; to consider the pros and cons of sickness absence (SA) with the patient; to determine the length and duration of SA requires; to determine the need for contact with others in health care; and to document statements, actions and plans according to common guidelines.

Physicians' sickness certification practice can be described in terms of handling patterns in professional practice, and includes tasks concerning disability evaluation.

Physicians have reported experiencing difficulties in issues concerning sickness certification, such as in assessments of work capacity. Further, there is research showing poor competence among physicians regarding sickness certification tasks due to a lack of knowledge of and training concerning guidelines.

So far, physicians' sickness certification practices seem only to have been studied to a limited extent. Since this is a common task and there are many actions taken to improve the processes involved, there is a need

of a systematically review what have been published.

19. * Participants/population.

Specify the participants or populations being studied in the review. The preferred format includes details of both inclusion and exclusion criteria.

Physicians undertaking sickness certification tasks.

20. * Intervention(s), exposure(s).

Give full and clear descriptions or definitions of the interventions or the exposures to be reviewed. The preferred format includes details of both inclusion and exclusion criteria.

Interventions regarding education, guidelines, guidelines adherence, and assessment for physicians' sickness certification practice.

21. * Comparator(s)/control.

Where relevant, give details of the alternatives against which the intervention/exposure will be compared (e.g. another intervention or a non-exposed control group). The preferred format includes details of both inclusion and exclusion criteria.

Control: another intervention or a non-exposed control group, concerning natural/quasi-experiments the control will be before/after measures.

22. * Types of study to be included.

Give details of the study designs (e.g. RCT) that are eligible for inclusion in the review. The preferred format includes both inclusion and exclusion criteria. If there are no restrictions on the types of study, this should be stated.

Inclusion:

RCTs and intervention studies including data on physicians and on possible effects of intervention(s) (including e.g., natural/quasi-experiments) on physicians' sickness certification competence and/or practices, presenting physician- and/or patient-related outcomes.

Studies published in scientific journals after peer-review in the English language in the years 2009-2020.

Intervention studies included in previous systematic literature reviews on physicians' sickness certification competence and/or practice, covering literature from before November 2002 and from January 2002 to August 2009, will also be included to capture all potential relevant studies.

Exclusion:

Studies with only health economic outcomes will not be included.

23. Context.

Give summary details of the setting or other relevant characteristics, which help define the inclusion or exclusion criteria.

Interventions for physicians' sickness certification competence and/or practice.

24. * Main outcome(s).

Give the pre-specified main (most important) outcomes of the review, including details of how the outcome is defined and measured and when these measurement are made, if these are part of the review inclusion criteria.

Interventions for physicians' sickness certification, as assessed by:

Patients' absence from work due to sickness or injury, their return to work, and related outcomes such as absenteeism, sick leave, or work resumption at different time points (during the period after the intervention).

Physicians' outcomes related to the intervention, primarily knowledge, competence and adherence (after the intervention).

* Measures of effect

Please specify the effect measure(s) for you main outcome(s) e.g. relative risks, odds ratios, risk difference, and/or 'number needed to treat.

As stated above.

25. * Additional outcome(s).

List the pre-specified additional outcomes of the review, with a similar level of detail to that required for main outcomes. Where there are no additional outcomes please state ‘None’ or ‘Not applicable’ as appropriate to the review

None.

* Measures of effect

Please specify the effect measure(s) for you additional outcome(s) e.g. relative risks, odds ratios, risk difference, and/or 'number needed to treat.

Relative risks of RTW/SA

26. * Data extraction (selection and coding).

Describe how studies will be selected for inclusion. State what data will be extracted or obtained. State how this will be done and recorded.

To facilitate the evaluation of relevance the free systematic reviews web application “Rayyan QCRI” will be used. A screening, at title and abstract level, will be conducted independently by two investigators to assess eligibility and compliance to the inclusion and exclusion criteria as well as for full-text assessment, as recommended. Blinding will be applied at title and abstract level of screening. Any discrepancies will be resolved by consulting a third reviewer to reach an agreement. The intervention studies identified in previous reviews will also be included. The selection of publications will be presented with a PRISMA (Preferred Reporting Items for Systematic Reviews and Meta-Analyses) flow chart. The full texts for selected publications will be retrieved, and relevant studies meeting the inclusion criteria for the systematic review will be read in full, and relevant information extracted. Both the descriptive data on study characteristics and results from analysis will be extracted.

List of data to be extracted: first author, publication (year), country, verbatim aim, study design, setting, follow-

up/year of inclusion, population- participation- and dropout rates with gender distributions, intervention components, the source where the data was retrieved from (registers, questionnaire, interviews), outcomes (all), direction of intervention effect, method for analysis, outcome measures, results.

Further, for studies with outcomes suitable for the meta-analysis, also effect estimates will be retrieved.

27. * Risk of bias (quality) assessment.

State which characteristics of the studies will be assessed and/or any formal risk of bias/quality assessment tools that will be used.

A modified checklist from the Swedish agency for health technology assessment and assessment of social services, will be used for the assessment of relevance and quality. Risk of bias assessment will be conducted independently by at least two researchers and disagreements will be addressed via discussion till consensus is reached.

28. * Strategy for data synthesis.

Describe the methods you plan to use to synthesise data. This **must not be generic text** but should be **specific to your review** and describe how the proposed approach will be applied to your data. If meta-analysis is planned, describe the models to be used, methods to explore statistical heterogeneity, and software package to be used.

Characteristics of the included studies according to the extracted data will be summarized in tables. Where appropriate, and subject to availability of suitable data, quantitative results will be drawn together in a narrative synthesis.

Subject to availability of data, a random effects meta-analysis will be conducted to estimate summary effect estimates for outcome data obtained from studies with reported similar outcome measures.

A summarised relative risk estimate with 95% confidence interval (CI) will be calculated for the dichotomous variables such as return to work, and we will calculate standardized mean difference (SMD) with 95% CI for the non-dichotomous variables: knowledge.

A meta-analysis will be conducted using Stata 12 and the results of this will be summarized in forest plots.

We will also perform the sensitivity analyses for the primary outcomes by excluding studies with high or unclear risk of bias. If sufficient information has not been provided, we will if possible contact the authors.

29. * Analysis of subgroups or subsets.

State any planned investigation of ‘subgroups’. Be clear and specific about which type of study or participant will be included in each group or covariate investigated. State the planned analytic approach.

The included studies will be grouped based on type of intervention, type of physician, type of outcome etc.

30. * Type and method of review.

Select the type of review, review method and health area from the lists below.

Type of review

Cost effectiveness
No

Diagnostic
No

Epidemiologic
No

Individual patient data (IPD) meta-analysis
No

Intervention
Yes

Meta-analysis
Yes

Methodology
No

Narrative synthesis
No

Network meta-analysis
No

Pre-clinical
No

Prevention
No

Prognostic
No

Prospective meta-analysis (PMA)
No

Review of reviews
No

Service delivery
No

Synthesis of qualitative studies
No

Systematic review
Yes

Other
No

Health area of the review

Alcohol/substance misuse/abuse
No

Blood and immune system
No

Cancer
No

Cardiovascular
No

Care of the elderly
No

Child health
No

Complementary therapies

No

COVID-19
No

Crime and justice
No

Dental
No

Digestive system
No

Ear, nose and throat
No

Education
No

Endocrine and metabolic disorders
No

Eye disorders
No

General interest
Yes

Genetics
No

Health inequalities/health equity
No

Infections and infestations
No

International development
No

Mental health and behavioural conditions
No

Musculoskeletal
No

Neurological
No

Nursing
No

Obstetrics and gynaecology
No

Oral health
No

Palliative care
No

Perioperative care
No

Physiotherapy
No

Pregnancy and childbirth
No

Public health (including social determinants of health)
Yes

Rehabilitation
No

Respiratory disorders
No

Service delivery
Yes

Skin disorders
No

Social care
Yes

Surgery
No

Tropical Medicine
No

Urological
No

Wounds, injuries and accidents
No

Violence and abuse
No

31. Language.

Select each language individually to add it to the list below, use the bin icon to remove any added in error.
English

There is an English language summary.

32. * Country.

Select the country in which the review is being carried out. For multi-national collaborations select all the countries involved.

Netherlands
Sweden

33. Other registration details.

Name any other organisation where the systematic review title or protocol is registered (e.g. Campbell, or The Joanna Briggs Institute) together with any unique identification number assigned by them. If extracted data will be stored and made available through a repository such as the Systematic Review Data Repository (SRDR), details and a link should be included here. If none, leave blank.

34. Reference and/or URL for published protocol.

If the protocol for this review is published provide details (authors, title and journal details, preferably in Vancouver format)

Add web link to the published protocol.

Or, upload your published protocol here in pdf format. Note that the upload will be publicly accessible.

No I do not make this file publicly available until the review is complete

Please note that the information required in the PROSPERO registration form must be completed in full even if access to a protocol is given.

35. Dissemination plans.

Do you intend to publish the review on completion?

Yes

Give brief details of plans for communicating review findings.?

As two publications in peer reviewed scientific journals; one regarding outcomes related to patients' sickness absence/return to work and one regarding physicians. Results will also be presented at national and international scientific conferences.

36. Keywords.

Give words or phrases that best describe the review. Separate keywords with a semicolon or new line. Keywords help PROSPERO users find your review (keywords do not appear in the public record but are included in searches). Be as specific and precise as possible. Avoid acronyms and abbreviations unless these are in wide use.

Sickness certification; Sick leave; Physicians' practice patterns; Intervention

37. Details of any existing review of the same topic by the same authors.

If you are registering an update of an existing review give details of the earlier versions and include a full bibliographic reference, if available.

Wahlstrom R, Alexanderson K. Swedish Council on Technology Assessment in Health Care (SBU). Chapter 11. Physicians' sick-listing practices. Scand J Public Health Suppl. 2004;63:222-55. doi:10.1080/14034950410021916.

Söderberg E, Lindholm C, Kärrholm J, Alexanderson K. Läkares sjukskrivningspraxis. En systematisk litteraturöversikt. [in Swedish] SOU 2010:107. Socialdepartementet, Sociala rådet.; 2010.

38. * Current review status.

Update review status when the review is completed and when it is published.New registrations must be ongoing.
Please provide anticipated publication date

Review_Ongoing

39. Any additional information.

Provide any other information relevant to the registration of this review.

The first review has been submitted, the second review is ongoing.

40. Details of final report/publication(s) or preprints if available.

Leave empty until publication details are available OR you have a link to a preprint. List authors, title and journal details preferably in Vancouver format.

Give the link to the published review or preprint.
